# Correspondence of perceived vs. objective proximity to parks and their relationship to park-based physical activity

**DOI:** 10.1186/1479-5868-6-53

**Published:** 2009-08-11

**Authors:** Kelsey J Lackey, Andrew T Kaczynski

**Affiliations:** 1 Department of Kinesiology, Kansas State University, Manhattan, KS, USA

## Abstract

**Background:**

Parks are key environmental resources for encouraging population-level physical activity (PA). In measuring availability of parks, studies have employed both self-reported and objective indicators of proximity, with little correspondence observed between these two types of measures. However, little research has examined how the degree of correspondence between self-reported and objectively-measured distance to parks is influenced by individual, neighborhood, and park-related variables, or which type of measure is more strongly related to physical activity outcomes.

**Methods:**

We used data from 574 respondents who reported the distance to their closest park and compared this with objective measurements of proximity to the closest park. Both indicators were dichotomized as having or not having a park within 750 m. Audits of all park features within this distance were also conducted and other personal characteristics and neighborhood context variables (safety, connectedness, aesthetics) were gleaned from participants' survey responses. Participants also completed detailed seven-day PA log booklets from which measures of neighborhood-based and park-based PA were derived.

**Results:**

Agreement was poor in that only 18% of respondents achieved a match between perceived and objective proximity to the closest park (kappa = 0.01). Agreement was higher among certain subgroups, especially those who reported engaging in at least some park-based PA. As well, respondents with a greater number of parks nearby, whose closest park had more features, and whose closest park contained a playground or wooded area were more likely to achieve a match. Having a ball diamond or soccer field in the closest park was negatively related to achieving a match between perceived and objective proximity. Finally, engaging in at least some park-based PA was not related to either perceived or objective proximity to a park, but was more likely when a match between and perceived and objective proximity occurred.

**Conclusion:**

Poor levels of correspondence were observed between self-reported and objective proximity to parks, but certain individual, neighborhood, and park variables increased the likelihood of a participant being aware of local parks. Future research should examine how people conceptualize parks and what urban and park planners can do to increase awareness and use of these community assets.

## Background

Physical activity (PA) is important for health and a growing body of evidence points to the influence of neighborhood and community environments on population-level rates of activity and inactivity [1-3]. Consequently, social ecological models are increasingly adopted in PA research and promotion that emphasize how numerous sectors and disciplines can contribute to improving active living behaviors [4,5]. Within this new paradigm, parks and open space are acknowledged as key behavior settings [6] that can facilitate or discourage PA depending on both their availability and design [7-9]. For example, in one study of travel diary data from more than 3000 children and teenagers in Atlanta, having at least one recreation or open space land use within 1 km from home was the built environment variable most consistently related to walking at least once over a two-day period and to walking greater than 0.5 miles per day [10]. Another study found that the number of features within a park was more important than its size or distance from residents in predicting use of the park for PA, and that certain features were more strongly-related to park-based PA than others [11].

A key issue in the emerging PA and built environment literature concerns how best to measure environmental correlates of PA [3,12]. Such debates include whether subjective or objective indicators are superior and the degree to which these different measurement modes correspond for the same objects being examined (e.g., nearest supermarket). Some recent studies have examined the level of agreement that exists when residents are asked to rate features of their neighborhoods versus when objective indicators such as observational audits or geographic information systems are used to characterize the same areas. These have generally reported low correspondence between subjective and objective indicators for most environmental features [13-17].

Likewise, the few studies that have included perceived and actual measures of park proximity have reported only poor to fair levels of agreement [14-16]. For example, Macintyre et al. (2008) calculated a kappa value of .095, which is considered poor [18], when comparing participants' reports of living with a half-mile of a park with GIS measurements of the presence or absence of a park within a half-mile buffer. Examining the degree of correspondence between subjective and objective indicators of park availability or proximity is important because it has been argued that people cannot make use of neighborhood resources or destinations for PA if they are not aware of them [13,16,19].

However, low levels of agreement may be attributable to the way parks have been treated in the majority of the literature to date. For example, almost all previous studies of park proximity, whether using subjective or objective measures, "have generally by default considered all parks and playgrounds to have the same elements and qualities, despite the awareness that they may differ substantially on these characteristics" [20]. This is also true of the few papers that have directly compared perceived and objective proximity to parks within the same study [14-17]. It has been noted that perceived distances and actual ability to predict distances may be influenced by the attractiveness of an end destination [21]. Thus, it may be that residents are unaware of nearby parks which are smaller or contain fewer features, but that greater awareness and greater correspondence with objective measurements (and consequently, perhaps, greater use for PA) may exist for parks that are larger or possess certain unique characteristics. To date, this has not been empirically examined in the parks and PA or broader built environment literature. Likewise, certain demographic (e.g., age), neighborhood (e.g., safety), or other park-related variables may influence the extent to which residents are aware of the available parks in their neighborhoods [17].

Finally, among the relatively few studies that have employed comparable measures of both perceived and objective environmental features in examining how each is related to physical activity, neither objective nor subjective measures have consistently shown a stronger relationship with study participants' PA levels [22-25]. With respect to parks specifically, one study found that self-reported greenness of one's neighborhood was significantly related to walking trips, but objective greenness was not [26]. Another reported that neither subjective nor objective indicators of proximity to a park were related to participants' meeting PA recommendations [23]. Mowen et al. [19] reported that perceived park proximity had significant direct and indirect relationships with reported park visitation, daily physical activity, and perceived health, whereas an objective measure of park proximity was related only to respondents' length of park stay. However, in spite of these few analyses, the majority of studies examining parks and PA have used subjective measures [27] and few others have included both objective and subjective indicators of park proximity in the same study to compare their relative importance in predicting PA. Better understanding whether (perceived) awareness, (objective) availability, or both is more important for predicting use of parks for PA may be valuable for knowing where to target efforts to promote PA through parks [13,17].

### Purpose

The purposes of this study were: i) to investigate the degree of correspondence between perceived and objective proximity to parks, including how this varies by individual, neighborhood, and park-related characteristics, and ii) to examine the association of both perceived and objective measures of park proximity - as well as a match between the two indicators - with both the likelihood of engaging in at least some neighborhood-based PA and at least some park-based PA.

## Methods

### Sampling and Data Collection

This project was part of a larger study on social ecological correlates of active living, with a particular emphasis on the role of parks in facilitating PA. The study took place in August 2006 within four neighborhoods in a medium-sized city in Ontario, Canada. Study packages consisting of a questionnaire and PA log booklet were distributed door-to-door by trained research assistants to 960 adults in households randomly-selected from city property lists, with completed materials received from 585 participants (60.9%). Almost all respondents indicated their perceived proximity to their closest park, one of the primary variables analyzed herein, and were thus eligible for inclusion in the present analyses (n = 574).

### Subjective Proximity to Parks

Subjective (or perceived) proximity to parks was measured by asking participants to indicate how long it would take to walk to the nearest park, with response options of 1-5 minutes, 6-10 minutes, 11-20 minutes, 21-30 minutes, and 31 or more minutes. This question was presented within a scale of 23 total neighborhood destinations (e.g., bank, supermarket) intended to capture the degree of neighborhood land use diversity, which has been employed in previous studies and has demonstrated good test-retest reliability [28,29]. As has been done in other studies, these estimates of time to walk to the closest park were translated into distances by multiplying by an average walking pace of 4.5 km/h [16]. Thus, the five time categories equated respectively to 0-375 m, 376-750 m, 751-1500 m, 1501-2250 m, and greater than 2250 m.

### Objective Proximity to Parks and Park Features

The procedures used for calculating objective distance to the closest park are described in greater detail elsewhere [30]. Briefly, the location and size of all parks (n = 54) within 800 m of the boundary of each of the four study neighborhoods were identified from geographic information systems and other databases provided by the study municipality. Distances from participants' addresses to the centroids of all parks were calculated and proximity to the closest park was categorized as 0-375 m, 376-750 m, 751-1500 m, 1501-2250 m, or greater than 2250 m to correspond with the subjective categories described above.

However, initial examination of the categorical data for both perceived and objective proximity showed very poor correspondence between the categories. For example, almost all participants (n = 512) perceived that the closest park was more than 750 m from their home, whereas the objective measurements showed that almost every participant had at least one park *within *750 m of their residence (n = 564). However, upon closer inspection, this discrepancy was somewhat understandable in that several of the 54 parks were very small (< 1 acre) and consisted of little more than nondescript open space. Thus, participants might not be blamed for either being unaware of these grass patches or for not conceptualizing them as a park. Consequently, the decision was made to remove parks for the purposes of further correspondence analyses if they satisfied all of the following criteria: a) less than 0.4 hectares (1 acre) in size, b) contained no significant features (e.g., trail, playground, ball diamond, water area), and c) not used for PA by participants during the study week (as described below). This resulted in 9 parks being removed from consideration, with 45 parks remaining that represented settings which participants would potentially and more reasonably identify as parks.

As part of the larger study, all parks were audited using the Environmental Assessment for Public Recreation Spaces (EAPRS) instrument. The EAPRS facilitates physical observation and inventory of the features of parks that may be related to PA [20]. In this paper, we examine how the total number of facilities in the closest park (out of 13) and the presence or absence of a select set of individual facilities (trail, wooded area, water area, playground, ball diamond, soccer field, basketball court) are related to the level of correspondence between participants' perceived and objective proximity to a park. We also examine the distance to the closest park, the size of the closest park, and the total number of parks within 750 m as predictors of correspondence.

### Measurement of Physical Activity and Other Correlates of Correspondence

Participants completed a 7-day PA log booklet in which they recorded the duration, intensity, and location as well as other details for each PA episode that was greater than ten minutes in length. Episode location descriptions recorded by participants as open-ended text were used to classify participants as engaging in a) at least some neighborhood-based PA (vs. none), defined as episodes that occurred (in whole or in part) outside of participants' homes on streets, in parks, or in other non-work areas within their neighborhood, and b) at least some park-based PA (vs. none), defined as episodes occurring specifically in a park within their neighborhood [30].

In addition to the log booklet, participants completed a questionnaire addressing a variety of personal and environmental correlates of physical activity. These included their gender, age (dichotomized as 18-39 years vs. 40+ years), marital status (married vs. other), education (college graduate vs. lower), body mass index calculated from self-reported height and weight (healthy vs. overweight/obese), the presence of children under 12 in the household, and whether they owned a membership to a fitness facility. We also measured self-efficacy for PA using an 11-item scale developed by Sallis et al. [31]. Finally, perceptions of neighborhood aesthetics and safety were measured using sub-scales of the Neighborhood Environment Walkability Scale [28] using 8 and 4 items, respectively, and a five-item scale was used to measure perceptions of neighborhood cohesion and trust [32]. The composite ratings of self-efficacy, safety, aesthetics, and cohesion were each dichotomized at their median value to categorize participants as high or low on these factors.

### Analyses

The degree of correspondence ("match") between perceived and objective measures of proximity to parks was assessed by dichotomizing both variables as the participant having a park or not having a park within 750 m (almost one-half mile) from home. The level of agreement between perceived and objective proximity was assessed using the kappa statistic, which takes into account agreement that occurs by chance [18]. Subsequently, using logistic regression, the odds of achieving a match were assessed in relation to several intrapersonal and environmental factors - a) the participant's gender, age, marital status, education level, family status, BMI, and self-efficacy, b) his or her perceptions of neighborhood safety, aesthetics, and cohesion, c) his or her level of neighborhood-based and park-based physical activity, and d) the distance to, size, number of total features, and presence of individual features within the closest park to his or her home, as well as the total number of parks within 750 m from home.

The second objective of the study was to examine how perceived and objective proximity to parks were related to PA outcomes. Two different PA outcomes were of interest: the participant engaging in a) any neighborhood-based PA, and b) any park-based PA. For each outcome, logistic regression analyses were conducted using three variables as predictors: perceived proximity to a park within 750 m, objective proximity to a park within 750 m, and the degree of correspondence between perceived and objective proximity (match vs. no match). All analyses controlled for age, gender, self-efficacy, and perceptions of neighborhood safety, aesthetics, and cohesion.

## Results

Table 1 shows descriptive characteristics for the study sample. Participants varied in age and were grouped into two categories - 18 to 39 years (39.9%) or 40 to 88 years (58.9%). Over half were female (55.4%) and the majority of respondents were either married or living in a common law relationship (77.2%). Two-thirds of respondents (66.0%) possessed at least a college level education, just over one-quarter (28.4%) lived in a household that contained a child under 12 years, and almost one-third (31.9%) owned a membership to a fitness facility. With respect to body mass index, approximately half (48.1%) were classified as being a healthy weight, while the other half (49.3%) were either overweight or obese. Most of the respondents (78.6%) reported participating in at least some neighborhood-based physical activity, whereas a much smaller percentage (25.1%) engaged in at least some park-based PA during the course of the study week.

**Table 1:** Sample Characteristics

Characteristic	N	%
Gender		
Male	251	43.7%
Female	318	55.4%
Missing	5	0.9%
Age		
18-39 years	229	39.9%
40-88 years	338	58.9%
Missing	7	1.2%
Marital Status		
Married or common law	443	77.2%
Not presently married	125	21.8%
Missing	6	1.0%
Education		
Less than college graduate	189	32.9%
College graduate or higher	379	66.0%
Missing	6	1.0%
Family Status		
Child under 12 in household	163	28.4%
No child under 12 in household	411	71.6%
Missing	0	0.0%
Body Mass Index		
Healthy weight	276	48.1%
Overweight or obese	283	49.3%
Missing	15	2.6%
Own a Fitness Facility Membership		
Yes	183	31.9%
No	383	66.7%
Missing	8	1.4%
Neighborhood-Based Physical Activity		
Any physical activity in neighborhood	451	78.6%
No physical activity in neighborhood	111	19.3%
Missing	12	2.1%
Park-Based Physical Activity		
Any physical activity in parks	144	25.1%
No physical activity in parks	430	74.9%
Missing	0	0.0%

### Perceived vs. Objective Proximity to Parks

Of the 574 participants, only 62 (11%) perceived that they lived within 750 m of a park, while 512 (89%) perceived that their closest park was more than 750 m away (Table 2). In contrast, using objective measurements, a large majority of participants (n = 501; 87%) had a park within 750 m, while 73 (13%) did not. The observed kappa value was 0.01 (indicating 'poor' agreement) and a match between perceived and objective proximity to the closest park (less than or greater than 750 m) was observed for 103 respondents (18%).

**Table 2:** Correspondence between Perceived and Objective Proximity to the Closest Park

	Objective Proximity			
	Closest park within 750 m		Closest park more than 750 m away	
Perceived Proximity	N	% of Total	N	% of Total
Closest park within 750 m	46	8.0%	16	2.8%
Closest park more than 750 m away	455	79.3%	57	9.9%

Several individual, neighborhood, and park-related variables were significantly related to increased or decreased odds of achieving a match between perceived and objective proximity (Table 3). A match was significantly less likely for participants over the age of 40 (OR = .48, CI = .21,.79), but significantly more likely for participants with at least a college education (OR = 1.43, CI = 1.06,1.75). Persons living in a household with a child younger than 12 years were almost twice as likely to correctly match perceived and objective measures of park proximity compared to those in households without young children (OR = 1.92, CI = 1.31,2.46). Individuals categorized as overweight or obese were significantly less likely to achieve a match (OR = .53, CI = .36,.81), as were individuals who owned a membership to a fitness facility (OR = .34, CI = .15,.76). Finally, participants who reported engaging in any park-based PA had significantly greater odds of correctly matching perceived and objective park proximity than participants who did not engage in park-based PA(OR = 2.15, CI = 1.36,2.88).

**Table 3:** Association of Individual, Neighborhood, and Park Variables with Perceived-Objective Park Proximity Match

Predictor of Perceived-Objective Match	OR	95% CI
*Individual variables*		
Gender (male)	0.94	(0.56,1.61)
Age (over 40 years)	0.48*	(0.21,0.79)
Marital status (married)	0.74	(0.41,1.33)
Education (college graduate)	1.43*	(1.06,1.75)
Family status (child <12 in house)	1.92*	(1.31,2.46)
BMI (overweight or obese)	0.53*	(0.36,0.81)
Self-efficacy for physical activity (high)	0.93	(0.53,1.60)
Any neighborhood physical activity	0.92	(0.48,1.76)
Any park-based physical activity	2.15*	(1.36,2.88)
Own a fitness facility membership	0.34*	(0.15,0.76)
		
*Neighborhood variables*		
Perceptions of safety (high)	0.98	(0.59,1.70)
Perceptions of aesthetics (high)	0.47*	(0.27,0.84)
Perceptions of cohesion (high)	1.35*	(1.09,1.67)
		
*Park variables*		
Number of parks within 750 m	1.07*	(1.03,1.10)
Size of closest park	0.99	(0.98,1.01)
Distance to closest park	1.17	(0.89,1.39)
Number of features in closest park	1.11*	(1.04,1.20)
Playground in closest park	1.24*	(1.07,1.55)
Trail in closest park	0.75	(0.42,1.33)
Wooded area in closest park	2.42*	(1.40,4.19)
Water area in closest park	1.15	(0.95,1.26)
Ball diamond in closest park	0.24*	(0.07,0.79)
Soccer field in closest park	0.26*	(0.08,0.85)
Basketball court in closest park	0.79	(0.38,1.66)

* odds ratio significant at the p < .05 level

While neighborhood-based PA was not a significant predictor of correctly matching park proximity (OR = .92, CI = .48,1.76), perceptions of certain neighborhood characteristics were significantly related to achieving a match (Table 3). People who perceived their neighborhoods as high in aesthetics had significantly lower odds of achieving a match (OR = .47, CI = .27,.84), while people who reported high neighborhood cohesion were significantly more likely to achieve a match (OR = 1.35, CI = 1.09,1.67).

Finally, certain park-related variables were significantly related to a participant achieving a match (Table 3). For example, a greater number of parks within 750 m was associated with greater odds of a match (OR = 1.07, CI = 1.03,1.10), as was having more features within the closest park to the participant (OR = 1.11, CI = 1.04,1.20). With respect to having individual features in the closest park from home, participants whose closest park contained a playground or wooded area were more likely to achieve a match (playground OR = 1.24, CI = 1.07,1.55; wooded area OR = 2.42, CI = 1.40,4.19). However, those whose closest park contained a ball diamond or soccer field were significantly less likely to achieve a match between perceived and objective park proximity (ball diamond OR = .24, CI = .07,.79; soccer field OR = .26, CI = .08,.85).

### Association of Perceived and Objective Proximity to Parks andPhysical Activity

Table 4 shows the results of models predicting the odds of engaging in neighborhood-based and park-based physical activity. Having a park within 750 m (measured objectively) was significantly related to greater odds of engaging in at least some neighborhood-based PA(OR = 1.12, CI = 1.01,1.25). Neither perceived proximity to a park within 750 m nor objective proximity to a park within 750 m was related to engaging in at least some park-based PA(perceived OR = 1.10, CI = .75,1.47; objective OR = .96, CI = .69,1.33). However, a match between perceived and objective proximity to a park within 750 m was significantly related to increased odds of engaging in at least some park-based PA (OR = 1.63, CI = 1.29,2.02).

**Table 4:** Association of Perceived and Objective Park Proximity with Neighborhood-Based and Park-Based Physical Activity

Proximity indicator	Neighborhood-Based Physical Activity		Park-Based Physical Activity	
	OR	95% CI	OR	95% CI
Perceived proximity	0.90	(0.47,1.72)	1.10	(0.75,1.47)
Objective proximity	1.12*	(1.01,1.25)	0.96	(0.69,1.33)
Match perceived/objective proximity	1.07	(0.85,1.26)	1.63*	(1.29,2.02)

* odds ratio significant at the p < .05 level

## Discussion

The primary purpose of this study was to examine the degree of correspondence between perceived and objectively-measured proximity to participants' closest park and how the level of correspondence varied according to several intrapersonal, neighborhood, and park-related variables. Achieving a match is important because people are unlikely to make use of PA resources of which they are unaware. Thus, it is important for park managers and other public health professionals to understand the public's awareness of facilities and services, what factors are associated with greater awareness, and how this familiarity might be enhanced.

In this study, we observed very poor agreement between perceived and objective proximity to parks, as indicated by a match for only 18% of participants and a kappa value of only 0.01. Other studies have reported similarly low levels of correspondence. For example, like our study, Kirtland et al. [17] reported a kappa value of 0.01 for survey and GIS measures for the presence of a park, playground, or sports field (grouped together) within 10 miles. Somewhat better, another study reported a kappa value of 0.39 ('fair' agreement) for a self-report measure of whether a park was within a 5-minute walk and whether one actually existed within 400 m of the respondent [14].

Other studies have more closely examined the direction or source of the mismatch. In Macintyre et al.'s [17] study of whether people reported and actually lived within a half-mile of a public green park, 62% (408/658) of respondents correctly predicted the presence or absence of a park within 0.5 miles - 355 (54%) said they lived within a half-mile of a park and they actually did, while 53 (8%) said they did not live near a park and GIS measurements confirmed this. However, for the remaining 250 people (38%) for which a match between self-reported and measured distance to a park was not observed, 51 (8%) believed there was no park within a half mile when there actually was, while 199 (30%) believed they lived within a half mile of a park but GIS measurements did not confirm their perceptions. Although that study achieved somewhat higher levels of agreement (62%), but a similarly poor kappa value (0.095), the source of disagreement was actually somewhat reversed compared to the present study. In Macintyre et al.'s study, a much greater percentage of those who incorrectly perceived the presence or absence of a nearby park were people who believed they lived within a half-mile of a park but really did not. In contrast, the present study reported a lack of awareness in that 79% of the sample (or 97% of the mismatched respondents) lived within 750 m of a park but stated they did not. Our results are similar to those of another study in which 76.5% of respondents overestimated the distance to their closest park (when given several categorical distance options), 15.3% correctly estimated the distance, and 8.2% underestimated the distance [16]. Another study reported a kappa value of 0.15 in which 81% of respondents to a survey said they lived within 0.5 miles of a park, whereas only 62% actually lived with 0.4 miles of a park [26]. Overall, the sources of disagreement have varied but past studies have generally reported poor correspondence between perceived and objective proximity to parks. These findings are consistent with conclusions from geography that people misestimate distances relative to their place of residence [33].

In this study, several variables were significant predictors of increased or reduced odds of achieving a match between perceived and objective proximity to parks. For example, with respect to socio-demographic characteristics, there was better correspondence for people who were younger, more educated, had children, and were a healthy weight. Further, a match was more likely among people who did not own a fitness facility membership and those who had engaged in at least-some park-based PA, with the latter finding being not surprising and perhaps suggesting a cyclical effect between park use and awareness. Similarly, a past study reported that more active people showed greater agreement for subjective and objective measures of proximity to recreation facilities, but not for many other environmental resources [15].

We also found that greater perceptions of neighborhood cohesion and lower perceptions of neighborhood aesthetics were associated with increased odds of achieving a match. Much research has suggested that constructs related to social capital have important implications for health [34-36]. In showing that perceptions of cohesion also increase awareness of neighborhood resources for PA, our findings add to the importance of urban planning that fosters improved bonds among residents and their communities. On the other hand, the finding about aesthetics is counterintuitive. It may be that persons living in aesthetically pleasing environments have less reason to seek out the environmentally-friendly confines of parks, but this and other hypotheses require exploration in future research. Finally, perceptions of neighborhood safety were unrelated to achieving a match. Past research has shown safety to be an important influence on PA behaviors [37-39], but such perceptions may not influence simple awareness of resources.

Finally, the level of correspondence between perceived and objective proximity was a function of several park-related variables. The number of parks within 750 m and the number of features in the closest park increased the odds of achieving a match, while the size of and distance to the closest park were not significant. These results are consistent with past research about important influences on park-based PA [11,30]. As well, having a playground or wooded area in the closest park was positively related to achieving a match, while having a ball diamond or soccer field was negatively related to achieving a match. It is, as yet, unclear whether the size (e.g., a large wooded area) or prominence (e.g., a colorful playground) of park features, or both, are more important for improving park awareness. However, some research has suggested that the intensity of park-based PA varies by activity area [40] and that certain park features more strongly influence the likelihood of PA occurring in parks [11]. Therefore, park awareness may be improved to the extent that the features in proximal parks are those that encourage use for PA or other active or non-active behaviors.

A secondary purpose of this study was to examine whether perceived or objective proximity to parks was more strongly related to a participant engaging in at least some neighborhood-based PA or at least some park-based PA. Objectively-measured proximity to a park was associated with neighborhood-based PA, but perceived proximity was not. It may be that having a park nearby (within 750 m in this case) facilitates an aesthetically and environmentally-pleasing setting that consciously or unconsciously encourages greater levels of activity in the vicinity of one's home. Another study found similar results in that the number of parks and the total area of parkland within 1 km of respondents' homes was related to greater levels of PA in the neighborhood [30]. Future research should examine the direct and indirect mechanisms by which proximal parks facilitate PA in different settings.

However, neither perceived nor objective proximity to a park within 750 m were related to engaging in at least some park-based PA. A recent study also reported that objectively-measured distance to the closest park was unrelated to engaging in park-based PA [30]. Likewise, with respect to perceptions, most studies have shown a general lack of awareness of nearby parks which would assumedly limit their use for PA by residents. Several studies have suggested that the content and design of parks may be more important than their proximity for encouraging PA [8,11]. For example, one study of parents reported that only 49% frequented the closest park to their starting destination, and instead, the majority chose to travel a significant distance (in some cases over 4 km) to attend parks with certain amenities [41]. Other research is starting to illuminate which features of parks are more conducive to promoting PA [11,40,42,43]. Park planners should keep in mind that neither perceived nor objective proximity may be the most important factors to consider when designing parks to encourage PA.

However, when a match occurred between perceived and objective proximity to a park within 750 m, a significant positive relationship was observed with the likelihood of engaging in park-based PA. Consequently, complementary efforts should be undertaken to both locate parks appropriately and promote their availability and features to residents [19]. A past study showed that even among people who had heard of several given parks, very few were knowledgeable about the specific features contained within them [44]. The budgets of park planners for promotional materials are often limited, but the present findings suggest that investments to communicate the whereabouts of parks and their specific features may pay off in terms of improved use for PA.

## Limitations

There were several important limitations to this study. First, we did not know or take into account participants' length of residence. This may or may not be a key determinant of correspondence but it is likely that awareness of proximal resources for PA would improve the longer one lives in a particular area. Future studies should investigate the veracity of this notion and potentially control for temporal variables or exclude respondents who have not lived in the neighborhood for a minimum period of time. As well, because of the quantitative survey data collection method, we do not know what people were considering to be a park when estimating the distance to their closest resource. As noted, we had concerns about such definitional issues for estimating correspondence and thus elected to exclude nine fairly inconsequential parks from the present analyses. This did not appear to improve the kappa value observed in our analysis compared to past studies, but did provide greater confidence that the 45 parks that we retained from the GIS database better reflected what residents would actually perceive as a park. However, future studies should use qualitative or other methods to investigate how residents perceive and define parks and what factors limit or enhance their awareness of these neighborhood resources [17].

Additionally, when examining the association of perceived and objective park proximity with two different PA indicators, we collected only seven days worth of PA data. The detailed data collected via the log booklets were invaluable for determining locations of PA episodes, but also limited the time frame we could expect residents to participate in the study. Finally, we did not examine residents' psychological attachment to parks or the meanings they attribute to neighborhood open spaces. Although we found that several personal, neighborhood, and park variables were significant predictors of correspondence, it may be that people for whom parks are more significant may demonstrate better judgments about distance.

## Conclusion

The findings of this study suggest that little correspondence exists between perceived and objectively-measured proximity to parks. Therefore, future researchers should be careful not to equate perceptions with actual distance. Just as importantly, given the lack of awareness about parks within 750 m, it is important to avoid assuming that actual distances are equivalent to perceptions [17] and that objective measures of proximity are necessarily superior. Despite these dismal correspondence findings, some encouraging results were observed in that participants who achieved a match between perceived and objective proximity to their closest park were more than one-and-a-half times more likely to engage in at least some park-based PA. Most past research has simply focused on perceived or objective proximity to parks without considering the inter-relationship between the two and their combined implications for active behaviors. Our finding suggests that both provision and promotion of parks are important for encouraging their use for PA. Further, the level of agreement was better for some sub-groups than others, suggesting that self-report measurements may be more accurate assessments of park proximity for certain people. Likewise, several park-related variables were related to increased or reduced odds of achieving a match between perceived and objective proximity. To our knowledge, this is the first study to examine correspondence according to the characteristics of nearby parks, but as research in this area accumulates, park planners would be wise to take note of the features that appear to improve awareness and use of neighborhood parks.

## Competing interests

The authors declare that they have no competing interests.

## Authors' contributions

Both authors participated in conceptualizing the paper, conducting analyses, and drafting and revising the manuscript. Both authors have read and approved the final manuscript.
